# Fermented barley and soybean (BS) mixture enhances intestinal barrier function in dextran sulfate sodium (DSS)-induced colitis mouse model

**DOI:** 10.1186/s12906-016-1479-0

**Published:** 2016-12-03

**Authors:** Jong Kyu Woo, Seungho Choi, Ju-Hee Kang, Dae Eung Kim, Byung-Serk Hurh, Jong-Eun Jeon, Sun Yeou Kim, Seung Hyun Oh

**Affiliations:** 1 Gachon Institute of Pharmaceutical Sciences, Gachon University, Incheon, 21999 Republic of Korea; 2 Laboratory of Developmental Biology and Genomics, College of Veterinary Medicine, Seoul National University, Seoul, 08826 Republic of Korea; 3 Research Institute, National Cancer Center, Goyang-si, Gyeonggi-do 410-769 Republic of Korea; 4 Sempio Fermentation Research Center, Osong, 363-954 Republic of Korea; 5 College of Pharmacy, Gachon University, 191 Hambakmoero, Yeonsu-gu, Incheon, 21999 Republic of Korea

**Keywords:** Dietary supplementation, Fermented foods, Intestinal bowel disease, Intestinal microbiota, Food supplements, Inflammatory bowel disease

## Abstract

**Background:**

Inflammatory bowel disease (IBD) is characterized by chronic or relapsing immune system activation and inflammation within the gastrointestinal tract. The lack of safety and efficacy of standard therapies, the use of food supplements for managing IBD is increasing, and many studies have reported that various food supplements provide many beneficial effects for the IBD.

**Methods:**

This study aimed to evaluate the anti-colitis effects of dietary supplementation with a fermented barley and soybean mixture (BS) on intestinal inflammation using a murine model of IBD. Female C57BL/6 mice were administered with either BS (100 and 200 mg/kg/day) or vehicle (PBS) control through oral gavages for 3 days and received 5% dextran sulfate sodium (DSS) drinking water to induce colitis. Mice body weight was measured every two days and disease activity index (DAI) score was determined on Day 15; mice were sacrificed and colons were analyzed by H & E staining and RT-PCR. We also measured intestinal barrier function in vitro using DSS-treated Caco-2 cells by assessing ZO-1 immunofluorescence staining and Western blotting and in vivo by measuring serum level of FITC-Dextran and by performing bacteria culture from mesenteric lymph nodes (MLN) extract. The gut microbiota was examined by real time PCR using fecal DNA.

**Results:**

We found that BS alleviated the severity of colitis in a DSS-induced colitis mouse model, and suppressed levels of pro-inflammatory cytokines in colonic tissue. Moreover, BS prevented epithelial barrier dysfunction, inducing an increase of tight junction protein levels in colonic tissues, BS also inhibited FITC-dextran permeability, and suppressed bacterial translocation to MLNs. In addition, BS increased the levels of *Lactobacilli* and *Bacteroides*, which have anti-inflammatory properties.

**Conclusion:**

Our study suggests that BS has protective roles against inflammatory bowel disease through changes in inflammatory activity, tight junction protein expression, and gut microbiota composition in DSS-induced colitis.

**Electronic supplementary material:**

The online version of this article (doi:10.1186/s12906-016-1479-0) contains supplementary material, which is available to authorized users.

## Background

Inflammatory bowel disease (IBD), including Crohn’s disease (CD) and ulcerative colitis (UC), is characterized by chronic inflammation of the gastrointestinal tract. Although the etiology of IBD remains poorly understood, IBD pathogenesis is reportedly associated with dysfunction of the intestinal epithelial barrier and alteration of intestinal microbiota [1].

Impaired epithelial barrier results in increased intestinal permeability to harmful bacteria and other antigens, leading to chronic immune response. Indeed, increased intestinal permeability is reported in IBD patients [2]. This indicates that dysfunction of epithelial barrier is likely an initial event before onset or recurrence of IBD.

Intestinal epithelial permeability is regulated by tight junction complex [3]. Several studies have observed structural abnormalities in tight junction complexes, including down-regulation of ZO-1, Occludin and Claudin-1, in IBD patients as a cause of altered intestinal permeability [4]. Therefore, modulation of intestinal permeability is a highly regarded target for novel therapeutic treatment against IBD.

In addition to alterations in tight junction complex integrity, gut microbiota is critical players in intestinal permeability. An imbalance between beneficial and pathogenic bacteria is involved in IBD pathogenesis [1].

Soybeans (*Glycine max*) are good sources of isoflavonoids (genistein and daidzein), which are bioactive components that have beneficial activity on health. They are known for anti-inflammatory, anti-cardiovascular, and anti-obesity activities [5]. Barley (*Hordeum vulgare*) is a major part of the diet as cereal. B-glucan, an active constituent of barley, has been reported to show anti-inflammatory and anti-cancer effects [6]. In our previous study, we developed a fermented barley and soybean mixture (BS) that increased production of β-glucan and isoflavonoids (genistein and daidzein). We showed that BS components produced significant protective effects against UVB-induced photoaging. Until now, it has been no report about the protective activity of BS on intestinal barrier function. In this study, we investigated whether BS protects the epithelial barrier and, if so, whether changes in tight junction protein expression and composition of microflora contribute to this effect. To answer these questions, mice with DSS-induced colitis were treated with BS, and epithelial permeability, tight junction protein expression, and fecal bacteria alteration were assessed.

## Methods

### Reagents

DSS (MW 36–50 kDa) was purchased from MP Biomedicals LLC (Santa Ana, CA, USA). DMEM, fetal bovine serum (FBS), penicillin (100 unit/mL) and streptomycin (100 μg/mL) were obtained from Welgene, Inc. (Daegu, Korea). The following antibodies were used in these studies: anti-ZO-1, anti-Occludin, anti-Claudin-1, IL-6 (Thermo Scientific, Grand Island, NY, USA), and GAPDH (Santa Cruz, CA, USA). Fermented barley and soybean (BS) were obtained from Sempio Fermentation Research Center (Osong, Korea). Barley (Hordeum vulgare) was cultivated by the agricultural Technology Center of Yeonggwang-gun, Jeollanam-do, and Republic of Korea. Soybean (Glycine max (L.) MERR) was supplied by Sempio Foods Company (Seoul, Korea). Voucher specimens were deposited in the herbarium at the R&D Center of Sempio Foods Company. Briefly, fermentation was performed using enzymatically hydrolyzed barley (40 g/L) and soybean (40 g/L) mediums. The medium was autoclaved and pH was adjusted to 7.0 with 2 M ammonia solution. The pre-cultured yeast, P. jadinii (KFCC 11487P), was inoculated into the enzymatically hydrolyzed barley and soybean. Fermentation was respectively conducted at 30 °C with shaking at 20 × g for 48 h in a 5 L bioreactor, and samples were dried and stored at −18 °C [7]. BS powder was dissolved in phosphate buffered saline (PBS) and then was stored at −20 °C.

### In vitro DSS treatment

Human colon carcinoma cell line (Caco-2) was obtained from the Korea Cell Line Bank (Seoul, Korea). Cells were grown at 37 °C in DMEM supplemented with 10% FBS, penicillin and streptomycin in a humidified atmosphere of 5% CO_2_. To test the effect of BS on DSS-treated Caco-2 cells, cells were seeded onto 12-well plates (SPL Life Science, Pocheon, Korea). After reaching 90–100% confluency, the Caco-2 cell monolayers were allowed to differentiate for an additional 14 days. Fully differentiated cell monolayers were incubated with or without 2% DSS in the absence or presence of 100, 200, and 400 μg/mL BS for 48 h. DSS was dissolved in culture media and filter-sterilized using a 0.45-μm filter [8–10].

### Immunofluorescence assay

Cells grown on glass coverslips and frozen tissues were fixed and permeabilized in methanol or acetone at −20 °C. Cells or tissues were incubated with primary antibodies overnight at 4 °C, followed by incubation with FITC-labeled secondary antibody for 1 h at room temperature. Sections were then mounted with mounting medium containing 4, 6-diamidino-2-phenylindole (DAPI) for nuclear counterstaining. Images were observed by fluorescence microscopy. FITC and DAPI images were taken from the same area.

### Western blot analysis

Whole cell and detergent-insoluble fractions were prepared as described previously [11]. Briefly, whole cell protein lysates and detergent-insoluble fractions were prepared in a modified RIPA buffer containing proteinase inhibitors and phosphatase inhibitors as described elsewhere [12]. Homogenates were spin down at 12,000 rpm at 4 °C for 20 min. Supernatants were collected for whole cell lysates and the pellets used as detergent-insoluble fractions. The pellets were dissolved in 0.1% SDS. Protein concentration of each samples was determined using BCA reagents (Thermo Scientific). Equivalent amounts of protein (20–80 μg) were loaded in 10% or 12% sodium dodecyl sulfate–polyacrylamide gel electrophoresis (SDS–PAGE) gels and transferred by blotting to polyvinylidene fluoride membranes. The blot was incubated with primary antibodies against human ZO-1, Occludin, Claudin-1, or GAPDH. After washing, the blot was incubated with HRP-conjugated secondary antibodies. The protein–antibody complexes were detected by Absignal (Abclone, Seoul, Korea) according to the manufacturer’s recommended protocol.

### Animal study

Six-week-old female C57BL/6 mice (weighing 20 ± 2 g) were received from the Orient Co. (a branch of Charles River Laboratories, Seoul, Korea). The mice were housed in a specific pathogen free (SPF) animal facility and acclimated under the conditions of 22 ± 2 °C, 40–60% relative humidity, and 12 h light/dark cycle for 7 days. Mice were divided into 4 groups of 5 mice each. The first group was vehicle-treated control and the second group was given drinking water with DSS only. The third and fourth groups of mice were treated with BS (100 and 200 mg/kg/day) through oral gavage for 3 days, then exposed to 5% DSS in their drinking water for 7 days to induce colitis. After DSS treatment, BS treatment groups were additionally administered BS for 4 days according to the experimental design. The study used the animal model to study the effects of fermented barley and soybean mixture during inflammation.

### In vivo permeability assay

In vivo permeability assay was performed to assess barrier function using fluorescein isothiocyanate dextran (FITC-D). For each experiment, mice were divided into 4 groups of 5 mice each. The first group was vehicle-treated control and the second group was given drinking water with DSS only. The third and fourth groups of mice were treated with BS (100 and 200 mg/kg/day) through oral gavage for 3 days, then exposed to 5% DSS in their drinking water for 7 days to induce colitis. Briefly, food and water were withdrawn for 4 h, and mice were inoculated with FITC-D by oral gavage (20 mg/kg). After 4 h, mice serum was collected and fluorescence intensity was measured (excitation, 492 nm; emission, 525 nm). Detection of viable bacteria in mesenteric lymph nodes (MLNs) represented bacterial translocation from the lumen to the MLNs. The MLNs of left colonic regions were removed aseptically and were put into eppendorf tubes with 0.1-mL sterilized PBS and tissues were homogenized by micro grinder (RPI, Mount Prospect, IL, USA). The homogenates were plated on blood agar (Thermo Fisher Scientific, Lenexa, KS, USA) and incubated for 48 h at 37 °C. The number of colonie was counted and the ratio of bacterial translocation was presented for percentages.

### Semi-quantitative RT-PCR and quantitative PCR (qPCR) with 16S rRNA for specific species

For semi-quantitative RT-PCR, 1 μg of RNA was used as a template for reverse-transcription using the Prime Script 1’st strand cDNA Synthesis kit (Takara; Kyoto, Japan). PCR was carried out with 20 ng of cDNA using a PCR pre-mixture (Takara). RT-PCR was performed to amplify genes using a cDNA template corresponding to gene-specific primer sets. The primer sequences used are as follows. Primer sequences are listed in the Additional file 1: Table S1. Fecal samples were collected before necropsy and immediately frozen in liquid nitrogen. Bacterial genomic DNA was extracted from fecal samples using a Wizard genomic DNA purification kit (Promega, Madison, WI, USA) according to the manufacturer’s instructions. The abundance of specific intestinal bacterial groups was measured by qPCR. Genus- or species-specific 16S rRNA gene primers were used as described previously [13]. Primer sequences are listed in the Additional file 1: Table S2. 16S rRNA of Eubacteria was used as a housekeeping gene.

### Statistical analysis

The results are analyzed by one-way analysis of variance (ANOVA) and differences were considered statistically significant at level of *p*-values < 0.05.

## Results

### The effect of BS on the structure of tight junction complexes in vitro

**Fig. 1:**
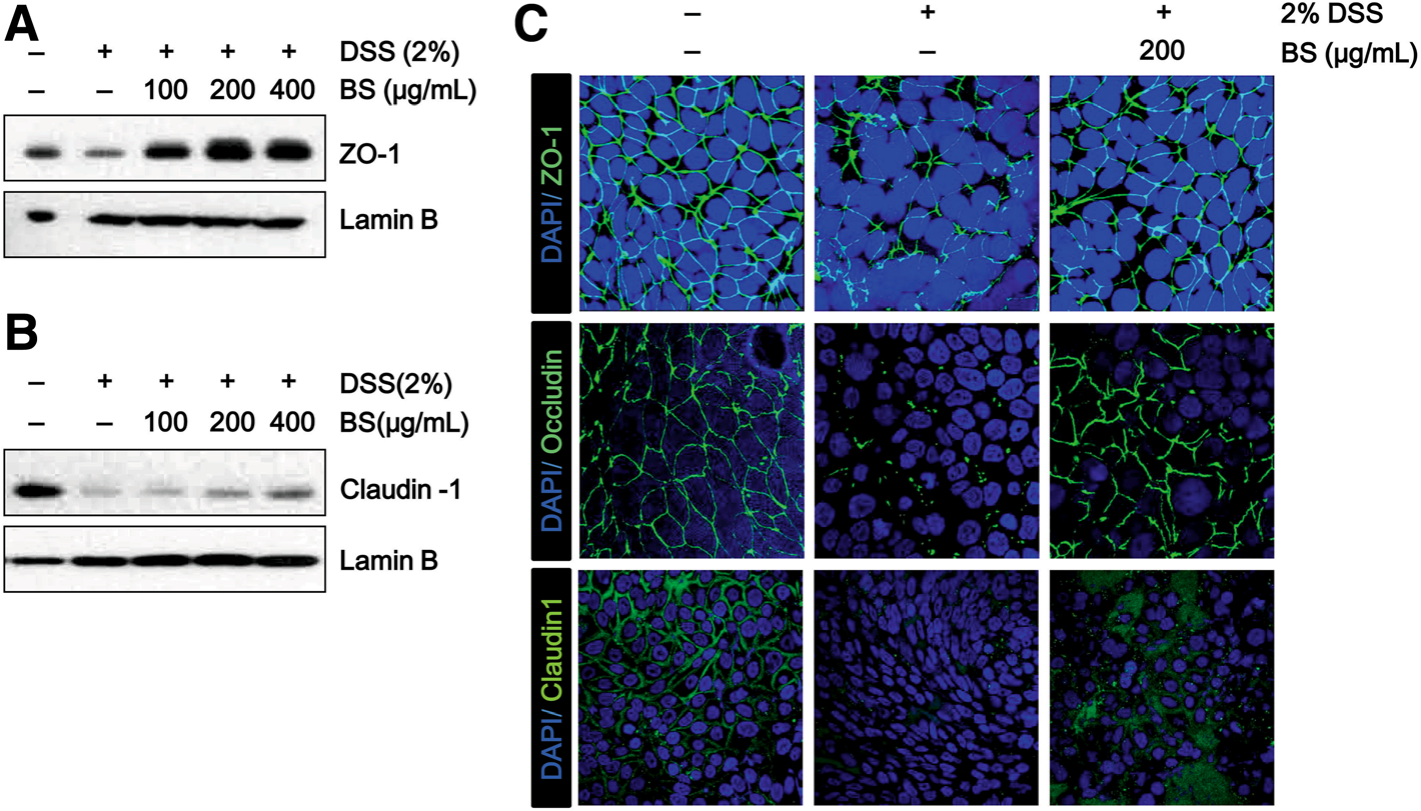
The effect of BS on the structure of tight junction complexes in vitro. (**a** and **b**) Immunoblot analysis of tight junction proteins in the detergent-insoluble fractions. **c** Immunofluorescence of ZO-1, Occludin, and Claudin-1 in Caco-2 cell monolayers incubated with 2% DSS in the absence or presence of indicated concentrations of BS for 48 h. Images were collected by confocal microscopy. Magnification x600

Since Caco-2 is an intestinal epithelial cell line and forms a monolayer when cultured to 100% confluence, it is broadly used as a model of the intestinal barrier [14]. To study the protective efficacy of BS in the epithelial barrier, the Caco-2 cell line was used. When the cells were incubated with DSS, the expression of tight junction proteins such as ZO-1, and caludin-1 was decreased. BS treatment dose-dependently recovered the loss of tight junction proteins in DSS-treated Caco-2 monolayer (Fig. 1a and b). Consistent with this, immunostaining with anti-ZO-1, Occludin and Claudin-1 showed that tight junction in DSS-induced monolayer was markedly disrupted, but treatment with BS protected tight junction complex from DSS-induced damage (Fig. 1c). These data suggest that BS might contribute maintenance of epithelial barrier integrity by preserving tight junction proteins. However, BS single treatment doesn’t enhanced expression of tight junction proteins (Additional file 2: Figure S2).

### BS prevents symptoms of DSS-induced colitis in mice

**Fig. 2:**
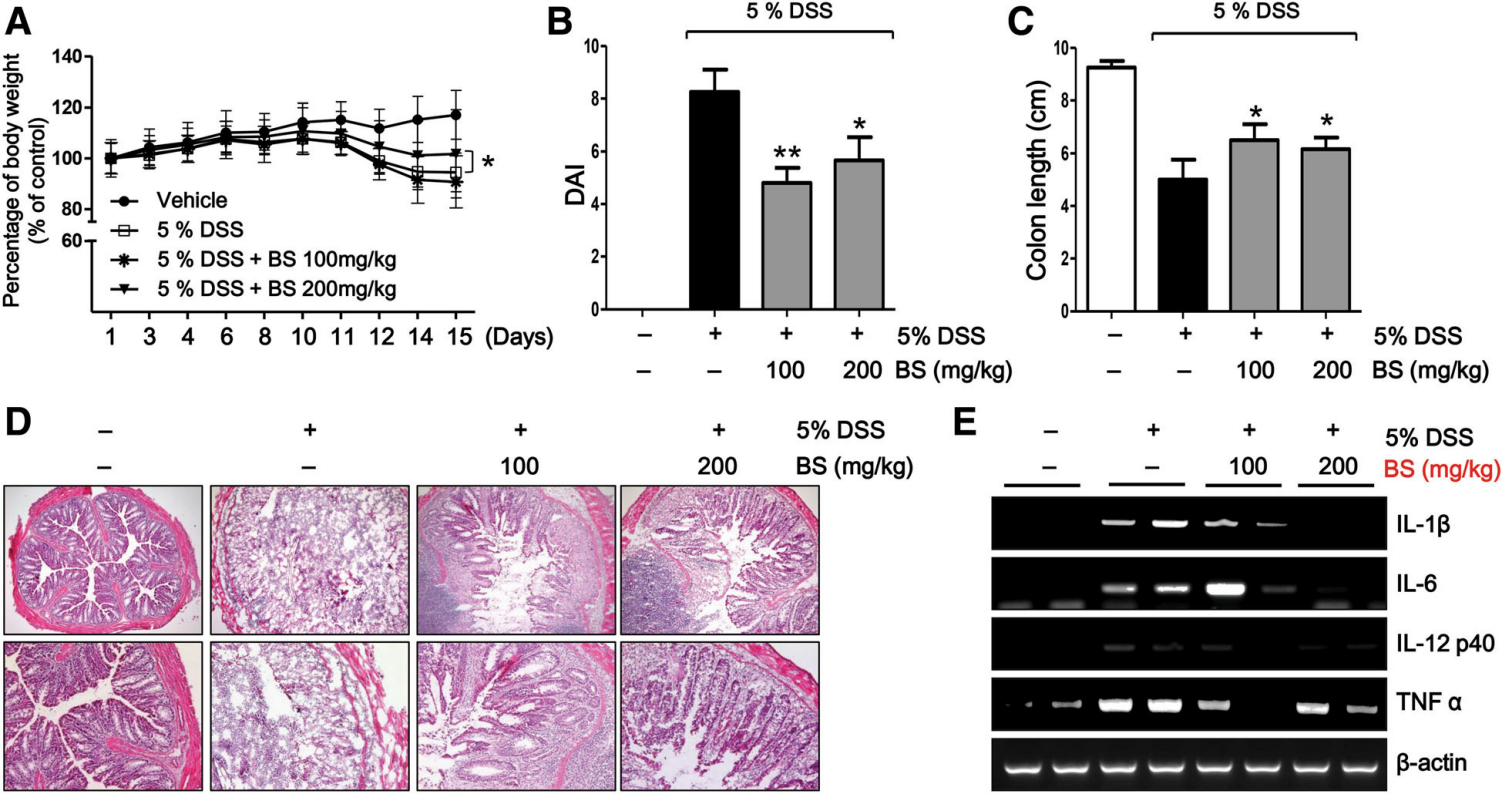
BS attenuated the progression of DSS-induced colitis. **a** Mice body weight was measured every other day for evaluation of BS efficacy on DSS-induced colitis, and was shown as percentage of weight change. **b** DAI and (**c**) colon length were evaluated. **d** Representative H&E stained histology from vehicle, 5% DSS, and BS-treated groups. Magnification: ×100 (*upper*), ×200 (*lower*) (**e**) Expression of pro-inflammatory cytokines in colon was assessed by semi-quantitative RT-PCR. Statistically differences were analyzed with one-way ANOVA. The data are presented as mean ± SD of triplicate experiments. *p < 0.05 and **p < 0.01 vs 5% DSS-treated group

To evaluate the therapeutic effect of BS on DSS-induced colitis, we investigated the ability of BS to ameliorate DSS-induced colitis by assessing body weight, disease activity index (DAI), and colon length and by performing histological analysis. We found that BS (200 mg/kg) treatment relieved the loss of body weight from day 12 to day 15, compared to the group treated with DSS only (Fig. 2a). Following induction of colitis, the BS treatment reduced DAI scores compared with only DSS treatment (Fig. 2b). In addition, while colon length was decreased in the DSS group compared with vehicle group, BS mitigated this shortening of colon length (Fig. 2c). Colon tissues were histologically analyzed to evaluate DSS-induced inflammation and epithelial damage. We found that DSS-treated mice exhibited acute colitis with severe inflammation and crypt damage. These changes were reduced by treatment with BS (Fig. 2d). In addition to macroscopic changes, production of pro-inflammatory cytokines is also important in the development of IBD [15]. Therefore, we investigated the effect of BS on pro-inflammatory cytokine production in the DSS-induced colitis model by using RT-PCR. We found that BS (200 mg/kg) significantly reduced DSS-induced mRNA levels of TNF-α, IL-1β, IL-6, and IL-12p40 in colonic tissues (Fig. 2e). At the same time, BS treated mouse showed a lower level of expression of the IL-6 in the inflamed colon (Additional file 2: Figure S4). These results indicate that the BS treatment diminished the severity of DSS-induced colitis. Moreover, nutrient supplementation leads to beneficial effects in a DSS-induced colitis model. We included LPS-induced colitis models to confirmed therapeutic activity of BS. We included Additional file 2: Figures S1 to demonstrate that activity of BS on LPS-induced colitis models. We found that BS (200 mg/kg) treatment relieved DAI scores compared with LPS treatment (Additional file 1: Figure S1A) and reduced LPS-mediated tissues damage (Additional file 1: Figure S1B).

### BS prevented DSS-induced disruption of tight junction complexes and loss of tight junction proteins

To investigate the protective effect of BS against DSS-induced disruption of tight junction, we examined the expression and organization of tight junction proteins including ZO-1, Occludin and Claudin-1 using immunofluorescence assay and Western blotting.

**Fig. 3:**
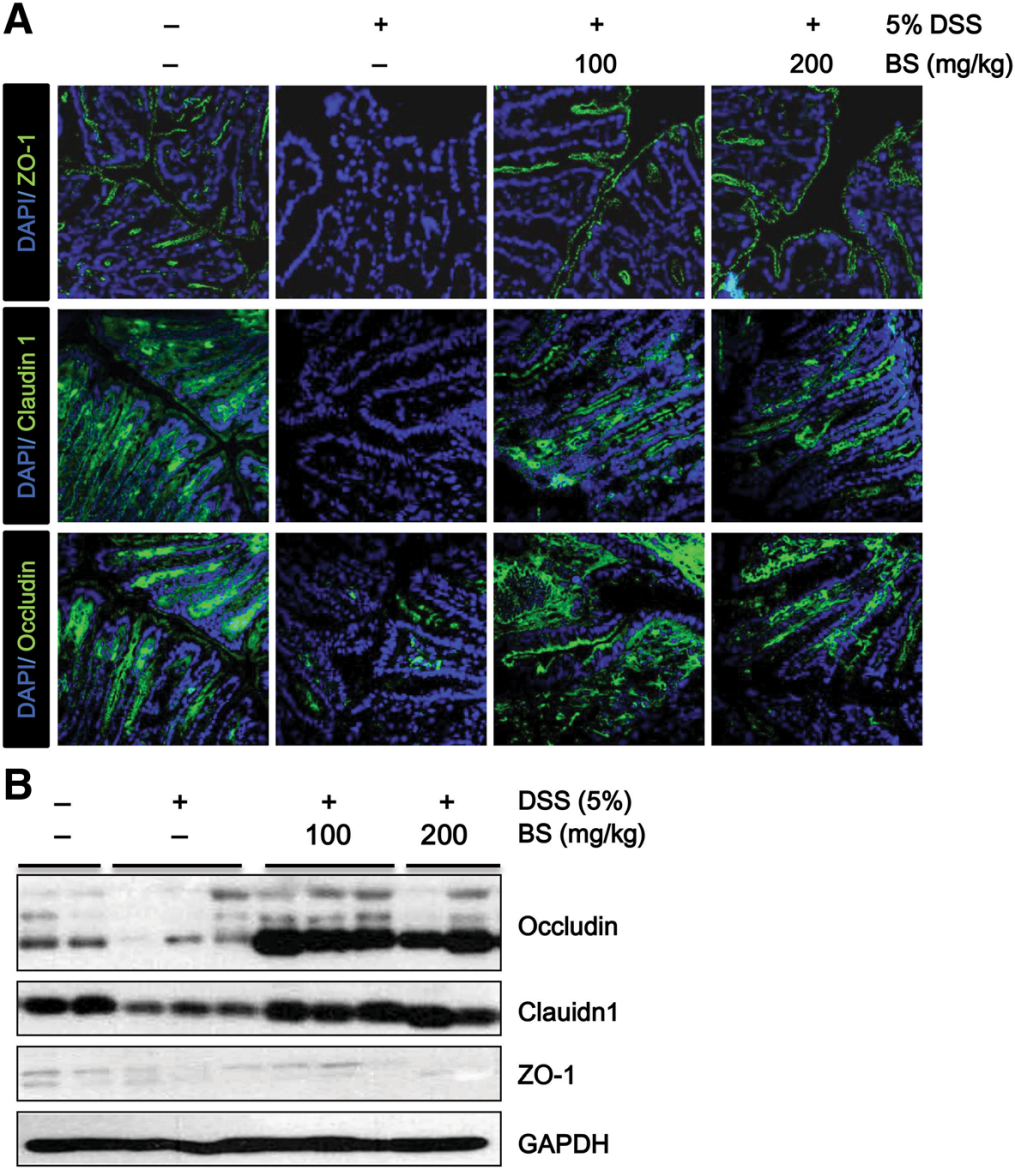
Effect of BS on colonic epithelial tight junctions proteins. **a** Expression of ZO-1, Occludin and Claudin-1 was examined by immunostaining using anti-zo-1 (*green*), anti-Occludin (*green*), and anti-Claudin-1 (*green*) antibodies in vehicle, DSS, and BS-treated groups. Magnification: ×600. **b** Expression of TJ proteins in the colon was assessed by Western blot

Immunofluorescence assays demonstrated considerable loss of ZO-1, Occludin, and Claudin-1 in tight junction complexes of the DSS-treated group and structural discontinuities in their architecture, in the inner lining of the colonic epithelium. Interestingly, BS treatment significantly prevented the loss of tight junction proteins, ZO-1, Occludin, and Claudin-1 in tight junction complexes of colonic epithelial cells (Fig. 3a).

To extend our observations of dramatic changes in tight junction proteins, we conducted Western blotting using colon tissues. Compared with the control group, there were significant reductions in the expression of Occludin and Claudin-1 in the DSS-treated group. In contrast, total protein level of ZO-1 remained unchanged. This indicates that the loss of Occludin and Claudin-1 at colonic epithelial tight junctions of DSS-treated mice was paralleled by quantitatively reduced protein expression (Fig. 3b). This might indicate a redistribution of existing proteins to the basolateral membrane. On the other hand, expression of tight junction proteins in BS-treated groups was significantly higher than in the DSS-treated group (Fig. 3b), suggesting that BS may play an important role in maintaining the integrity of tight junction complexes.

### BS prevented increased colonic epithelial permeability in DSS-induced colitis

**Fig. 4:**
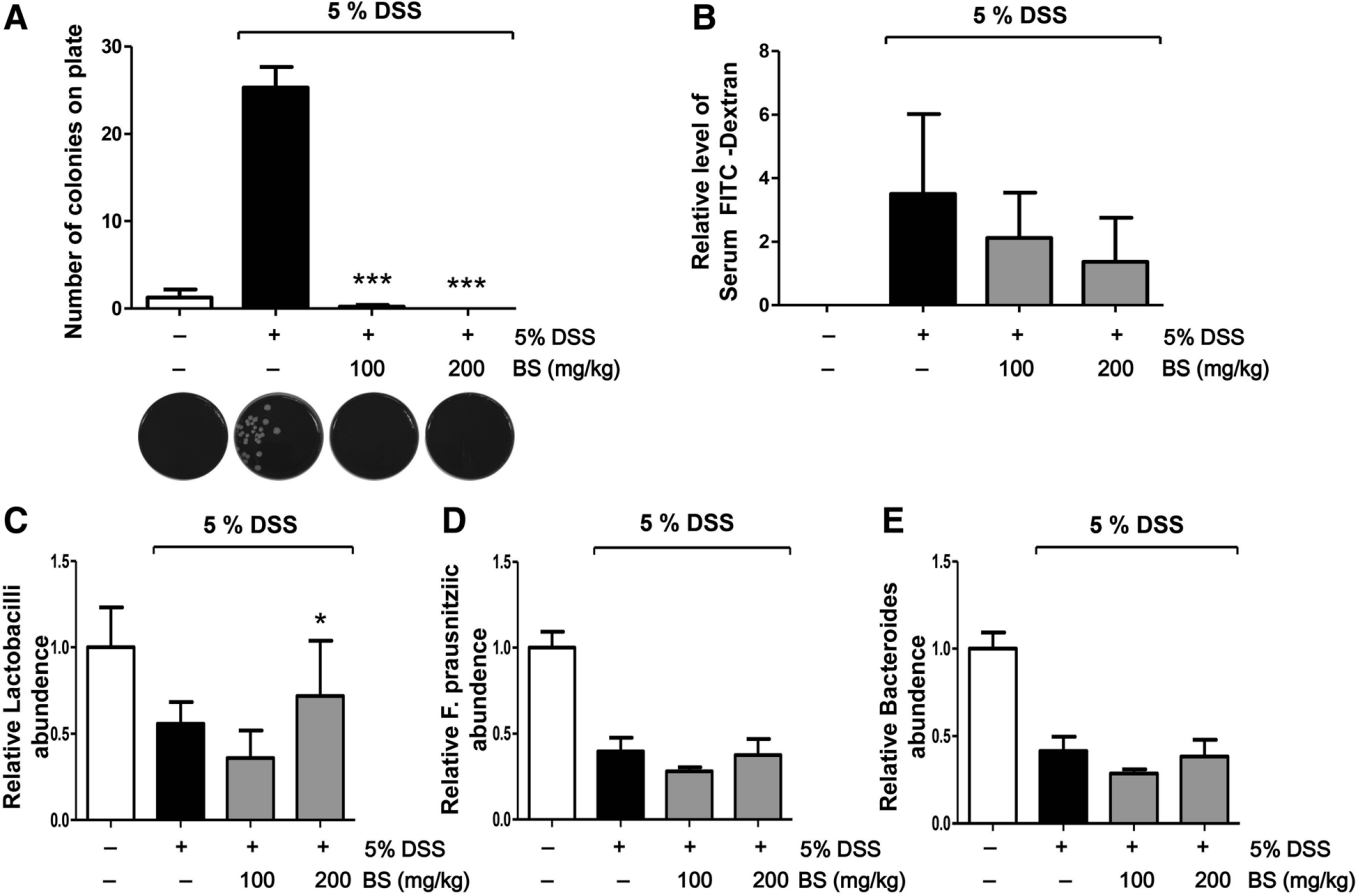
BS suppressed epithelial permeability and increased microbiota quantity in DSS-induced colitis model. **a** colony number of viable bacteria in MLNs, (**b**) Relative level of serum FITC-D, (**c**-**e**) fecal microbiota composition were shown in vehicle, DSS, and BS-treated groups; (**c**) *Lactobacilli*, (**d**) *Faecalibacterium prausnitzii*, and (**e**) *Bacteroides*. Statistically differences were analyzed with one-way ANOVA. The data are presented as mean ± SD of triplicate experiments. *p < 0.05 and ***p < 0.01 vs 5% DSS treated group

To determine the effect of BS on epithelial permeability, we analyzed intestinal permeability in DSS-induced colitis model. To that end, the level of bacterial translocation was measured (Fig. 4a). Compared with control group, a significant increase of bacterial translocation into the MLNs was observed in the DSS-treated group. Interestingly, the bacterial translocation was completely prevented by administration of BS. We also measured FITC-D permeability in order to determine if BS treatment prevented disruption of epithelial barrier function induced by DSS treatment (Fig. 4b). Mice in DSS-treated group had elevated levels of FITC-D in serum, compared with those in the control group. Administration of BS reduced FITC-D serum levels compared with that in the DSS-treated group. These results show that BS decreased epithelial permeability, suggesting that BS treatment prevented gut leakiness to a greater extent.

### BS regulated composition of fecal microflora in DSS-induced colitis mouse model

Gut microbiota are increasingly recognized as important players in intestinal permeability. The balance between beneficial and pathogenic bacteria plays a central role in the mucosal immune response in IBD. We investigated whether BS treatment changes the composition of microflora through analysis of feces (Fig. 4c-e). In this study, we observed that the quantity of bacteria associated with anti-inflammatory responses, such as *Bacteroides spp*., *Faecalibacterium prausnitzii*, and *Lactobacillus spp*., was diminished in fecal samples from the DSS-treated group compared with control group. Although contents of *Bacteroides* and *Faecalibacterium prausnitzii spp.* did not change, BS treatment prevented the decrease of *Lactobacillus spp*., suggesting that BS might contribute to the improvement of gut immune and barrier function by altering intestinal microbiota, which play a significant role in anti-inflammation.

## Discussion

Although some medicines such as 5-aminosalicylic acid have been developed to treat IBD, they are not effective enough to cure it. Moreover, most of the drugs currently used to treat IBD have untoward effects. Therefore, there is a growing need to develop efficient and safe treatments for IBD. Consequently, traditional medicinal plants, herb, vegetables and their secondary metabolites are considered to be alternative treatment strategies for IBD [16].

Many studies have reported that various food supplements provide many beneficial effects in the gut. β-glucan, produced by barley and oat, is known to have anti-inflammatory properties [17]. Dietary β-glucan is also effective to treat IBD [18, 19]. Genistein and daidzein, the active isoflavonoids mainly found in soybean, are also known to have anti-inflammatory effects in a colitis-induced mouse model [20, 21]. In addition, genistein has protective effects on intestinal barrier function by regulating tight junction [22]. Although these natural products contain many beneficial bioactive substances, there are some limits in terms of efficacy. Several studies have demonstrated that fermentation of the natural products improved biological activities [23]. Therefore, in order to enhance the beneficial effects of soybean and barley, we increased the activity of β-glucans, daidzein and genistein in barley and soybean by yeast fermentation.

Recently, experimental and clinical studies suggest that disruption and reassembly of tight junction is an important determinant of IBD [24, 25]. Our data show that BS treatment prevents DSS-induced redistribution or loss of tight junction proteins like ZO-1, Occludin, and Claudin-1 in Caco-2 monolayer.

To elucidate the anti-colitis potency of BS in IBD, we used a DSS-induced colitis model. Our findings show that BS suppresses symptoms of DSS-induced colitis such as body weight loss, colon length shortening, bloody feces and stool consistency. BS treatment also reduced the DSS-induced expression of anti-inflammatory cytokines such as TNF-α, IL-1β, IL-6, IL-12p40. The reduction in pro-inflammatory cytokines by BS may either be due to its direct suppressive effect on the expression of these pro-inflammatory cytokines or the indirect effect on epithelial barrier function. Once the epithelial barrier is damaged, foreign luminal antigenic products are able to cross the epithelial barrier and activate the immune system. Therefore, in addition to anti-inflammatory effects, we investigated the effect of BS on epithelial barrier function in DSS-induced colitis.

Consistent with the in vitro experiment, we showed that BS administration recovered expression or distribution of tight junction proteins, ZO-1, Occludin, and Claudin-1, in DSS-induced colitis. Furthermore, BS reduced epithelial barrier permeability in DSS-induced colitis, as demonstrated by suppression of FITC-D uptake and bacterial translocation into MLN. Our data showed that BS might have a protective effect on barrier integrity by maintaining the expression of tight junction proteins, thereby reducing the severity of colitis.

Accumulating evidence indicates that commensal bacteria are responsible for various physiological functions and are associated with immune-enhancing effects in the gut [26]. Several studies have suggested a critical role of microbial imbalances in the pathogenesis of IBD [27]. In IBD patients, the quantity of commensal bacteria in the intestine is reduced, and the diversity of the microbiota is also altered [1]. It was reported that *Bacterioidetes*, *Lactobacillus spp*. and *Faecalibacterium prausnitzii* are reduced in IBD patients [28]. Therefore, we analyzed the change of intestinal bacteria composition by using fecal DNA. We observed that *Lactobacillus spp*. was reduced in DSS-treated group, but BS administration prevented alteration of the bacterial composition.

## Conclusions

In summary, our data suggest that BS is effective for the amelioration of DSS-induced acute colitis, recovering epithelial barrier function and regulating the gut microbiota community.

## Additional files

Additional file 1: Table S1. Primer sets used for semi-quantitative PCR of cytokines. **Table S2.** Primer sets used for quantitative PCR of 16S rRNAof specific species or genus. (PDF 105 kb)
Additional file 2: Figure S1. BS promotes recovery from LPS-induced colitis and inflammation in mice. (A) DAI of LPS (LPS, 5 mg/kg of mouse by Intra-peritoneal injection) treated mice were scored. (B) Representative H&E stained histology from Control and BS-treated groups and statistical results. Mean value was significantly different from that of the LPS treated control group (**P* < 0.05). BS, Fermented barley and soybean, DAI, disease activity index. LPS, Lipopolysaccharide. **Figure S2.** Basal intestinal barrier function in BS treatment. (A) Immunofluorescence of ZO-1 in Caco-2 cell monolayers incubated with BS for 48 h and images were collected by confocal microscopy. (B) RNA levels of ZO-1, Claudin1, and Occludinin BS treated Caco-2 cells measured by semi-quantitative RT-PCR. (C) BS treated mouse colon tissues were used to determine ZO-1, Claudin1 and Occludindistribution by immune fluorescence staining and images were collected by confocal microscopy. BS, Fermented barley and soybean. **Figure S3.** Effect of BS on the suppression of NF-κBactivity. (A) Western blot analysis of p65 nuclear trans-localization levels in the protein fractions of RAW 264.7 lysates. (B) The effects of BS on a NF-κBreporter assay in HEK 293 cells that were activated by the addition of LPS. The luciferase activity was measured as relative light intensity, using a plate reader in the luminescence mode. BS, Fermented barley and soybean, LPS, Lipopolysaccharide. **Figure S4.** Immunohistochemicalstaining of IL-6 in sections from BS treated mouse colon. BS, Fermented barley and soybean. (PDF 563 kb)

## Abbreviations

BS: Fermented barley and soybean
CD: Crohn’s disease
DSS: Dextran sulfate sodium
FITC-D: Fluorescein isothiocyanate dextran
IBD: Inflammatory bowel disease
MLN: Mesenteric lymph nodes

## References

[1] Sartor RB, Mazmanian SK (2012). Intestinal Microbes in Inflammatory Bowel Diseases. Am J Gastroenterol Suppl.

[2] Gerova VA, Stoynov SG, Katsarov DS, Svinarov DA (2011). Increased intestinal permeability in inflammatory bowel diseases assessed by iohexol test. World J Gastroenterol.

[3] Laukoetter MG, Bruewer M, Nusrat A (2006). Regulation of the intestinal epithelial barrier by the apical junctional complex. Curr Opin Gastroen.

[4] Kucharzik T, Walsh SV, Chen J, Parkos CA, Nusrat A (2001). Neutrophil transmigration in inflammatory bowel disease is associated with differential expression of epithelial intercellular junction proteins. Am J Pathol.

[5] Lee Y-K, Park OJ (2013). Soybean isoflavone genistein regulates apoptosis through NF-κB dependent and independent pathways. Exp Toxicol Pathol.

[6] Ahmad A, Anjum FM, Zahoor T, Nawaz H, Dilshad SM (2012). Beta glucan: a valuable functional ingredient in foods. Crit Rev Food Sci Nutr.

[7] Lee S, Kim JE, Suk S, Kwon OW, Park G, Lim TG, Seo SG, Kim JR, Kim DE, Lee M (2015). A fermented barley and soybean formula enhances skin hydration. J Clin Biochem Nutr.

[8] Araki Y, Sugihara H, Hattori T (2006). In vitro effects of dextran sulfate sodium on a Caco-2 cell line and plausible mechanisms for dextran sulfate sodium-induced colitis. Oncol Rep.

[9] Zhao H, Zhang H, Wu H, Li H, Liu L, Guo J, Li C, Shih DQ, Zhang X (2012). Protective role of 1,25(OH)2 vitamin D3 in the mucosal injury and epithelial barrier disruption in DSS-induced acute colitis in mice. BMC Gastroenterol.

[10] Chang KW, Kuo CY (2015). 6-Gingerol modulates proinflammatory responses in dextran sodium sulfate (DSS)-treated Caco-2 cells and experimental colitis in mice through adenosine monophosphate-activated protein kinase (AMPK) activation. Food Funct.

[11] Noda S, Tanabe S, Suzuki T (2013). Naringenin enhances intestinal barrier function through the expression and cytoskeletal association of tight junction proteins in Caco-2 cells. Mol Nutr Food Res.

[12] Oh SH, Woo JK, Yazici YD, Myers JN, Kim WY, Jin Q, Hong SS, Park HJ, Suh YG, Kim KW (2007). Structural basis for depletion of heat shock protein 90 client proteins by deguelin. J Natl Cancer Inst.

[13] Wang H, Xue YS, Zhang HY, Huang Y, Yang G, Du M, Zhu MJ (2013). Dietary grape seed extract ameliorates symptoms of inflammatory bowel disease in IL10-deficient mice. Mol Nutr Food Res.

[14] Sambuy Y, De Angelis I, Ranaldi G, Scarino ML, Stammati A, Zucco F (2005). The Caco-2 cell line as a model of the intestinal barrier: influence of cell and culture-related factors on Caco-2 cell functional characteristics. Cell Biol Toxicol.

[15] Neurath MF (2014). Cytokines in inflammatory bowel disease. Nat Rev Immunol.

[16] Ke F, Yadav PK, Ju LZ (2012). Herbal Medicine in the Treatment of Ulcerative Colitis. Saudi J Gastroentero.

[17] Akramiene D, Kondrotas A, Didziapetriene J, Kevelaitis E (2007). Effects of beta-glucans on the immune system. Medicina.

[18] Ye MB, Bak JP, An CS, Jin HL, Kim JM, Kweon HJ, Choi DK, Park PJ, Kim YJ, Lim BO (2011). Dietary beta-glucan regulates the levels of inflammatory factors, inflammatory cytokines, and immunoglobulins in interleukin-10 knockout mice. J Med Food.

[19] Santos MDN, Magalhaes JED, Castro LSEPW, Pinheiro TD, Sabry DA, Nobre LTDB, Lima JPMS, Baseia IG, Leite EL (2014). Effect of Glucans from Caripia montagnei Mushroom on TNBS- Induced Colitis. Int J Mol Sci.

[20] Hamalainen M, Nieminen R, Vuorela P, Heinonen M, Moilanen E (2007). Anti-inflammatory effects of flavonoids: genistein, kaempferol, quercetin, and daidzein inhibit STAT-1 and NF-kappaB activations, whereas flavone, isorhamnetin, naringenin, and pelargonidin inhibit only NF-kappaB activation along with their inhibitory effect on iNOS expression and NO production in activated macrophages. Mediators Inflamm.

[21] Seibel J, Molzberger AF, Hertrampf T, Laudenbach-Leschowski U, Diel P (2009). Oral treatment with genistein reduces the expression of molecular and biochemical markers of inflammation in a rat model of chronic TNBS-induced colitis. Eur J Nutr.

[22] Suzuki T, Hara H (2011). Role of flavonoids in intestinal tight junction regulation. J Nutr Biochem.

[23] Lee TH, Do MH, Oh YL, Cho DW, Kim SH, Kim SY (2014). Dietary fermented soybean suppresses UVB-induced skin inflammation in hairless mice via regulation of the MAPK signaling pathway. J Agric Food Chem.

[24] Antoni L, Nuding S, Wehkamp J, Stange EF (2014). Intestinal barrier in inflammatory bowel disease. World J Gastroenterol.

[25] Su L, Shen L, Clayburgh DR, Nalle SC, Sullivan EA, Meddings JB, Abraham C, Turner JR (2009). Targeted epithelial tight junction dysfunction causes immune activation and contributes to development of experimental colitis. Gastroenterology.

[26] Tlaskalova-Hogenova H, Stepankova R, Kozakova H, Hudcovic T, Vannucci L, Tuckova L, Rossmann P, Hrncir T, Kverka M, Zakostelska Z (2011). The role of gut microbiota (commensal bacteria) and the mucosal barrier in the pathogenesis of inflammatory and autoimmune diseases and cancer: contribution of germ-free and gnotobiotic animal models of human diseases. Cell Mol Immunol.

[27] Martin R, Miquel S, Ulmer J, Kechaou N, Langella P, Bermudez-Humaran LG (2013). Role of commensal and probiotic bacteria in human health: a focus on inflammatory bowel disease. Microb Cell Fact.

[28] Matsuoka K, Kanai T (2015). The gut microbiota and inflammatory bowel disease. Semin Immunopathol.

