# Supplementary material for: Fermented barley and soybean (BS) mixture enhances intestinal barrier function in dextran sulfate sodium (DSS)-induced colitis mouse model
Source: BMC Complement Altern Med. 2016 Dec 3;16:498. doi: 10.1186/s12906-016-1479-0 (PMC5135811; doi:10.1186/s12906-016-1479-0)
Supplement: Additional file 1: Table S1. — Primer sets used for semi-quantitative PCR of cytokines. Table S2. Primer sets used for quantitative PCR of 16S rRNAof specific species or genus. (PDF 105 kb) [file 12906_2016_1479_MOESM1_ESM.pdf]

Supplementary Table S1

Supplementary Table S1. Primer sets used for semi-quantitative PCR of cytokines

Gene Name	Product Size	Sequence (5' to 3')
**IL-1β**	282bp	GCC TTG GGC CTC AAA GGA AAG AAT C
		GGA AGA CAC AGA TTC CAT GGT GAA G
**IL-6**	155bp	TGG AGT CAC AGA AGG AGT GGC TAA G
		TCT GAC CAC AGT GAG GAA TGT CCA C
**IL-12 p40**	201bp	GTC CTC AGA AGC TAA CCA TC
		TTT CCA GAG CCT ATG ACT CC
**TNFα**	258bp	ATA GCT CCC AGA AAA GCA AGC
		CAC CCC GAA GTT CAG TAG ACA
**β- actin**	349bp	TGG AAT CCT GTG GCA TCC ATG AAA C
		TAA AAC GCA GCT CAG TAA CAG TCC G

Supplementary Table S2

Supplementary Table S2. Primer sets used for quantitative PCR of 16S rRNA of specific species or genus

Target organism	Primer set	Product size	Sequence (5’ to 3’)
**Eubacteria**	UniF340	210	ACT CCT ACG GGA GGC AGC AGT
	UniR514		ATT ACC GCG GCT GCT GGC
**Lactobacillus**	LabF362	315	AGC AGT AGG GAA TCT TCC A
	LabR677		CAC CGC TAC ACA TGG AG
**Bacteroides**	BactF285	53	GGT TCT GAG AGG AGG TCC C
	UniR338		GCT GCC TCC CGT AGG AGT
**Faecalibacterium prausnitzii**	Fprau223F	199	GAT GGC CTC GCG TCC GAT TAG
	Fprau420R		CCG AAG ACC TTC TTC CTC C
