# Supplementary material for: Fermented barley and soybean (BS) mixture enhances intestinal barrier function in dextran sulfate sodium (DSS)-induced colitis mouse model
Source: BMC Complement Altern Med. 2016 Dec 3;16:498. doi: 10.1186/s12906-016-1479-0 (PMC5135811; doi:10.1186/s12906-016-1479-0)
Supplement: Additional file 2: Figure S1. — BS promotes recovery from LPS-induced colitis and inflammation in mice. (A) DAI of LPS (LPS, 5 mg/kg of mouse by Intra-peritoneal injection) treated mice were scored. (B) Representative H&E stained histology from Control and BS-treated groups and statistical results. Mean value was significantly different from that of the LPS treated control group (*P < 0.05). BS, Fermented barley and soybean, DAI, disease activity index. LPS, Lipopolysaccharide. Figure S2. Basal intestinal barrier function in BS treatment. (A) Immunofluorescence of ZO-1 in Caco-2 cell monolayers incubated with BS for 48 h and images were collected by confocal microscopy. (B) RNA levels of ZO-1, Claudin1, and Occludinin BS treated Caco-2 cells measured by semi-quantitative RT-PCR. (C) BS treated mouse colon tissues were used to determine ZO-1, Claudin1 and Occludindistribution by immune fluorescence staining and images were collected by confocal microscopy. BS, Fermented barley and soybean. Figure S3. Effect of BS on the suppression of NF-κBactivity. (A) Western blot analysis of p65 nuclear trans-localization levels in the protein fractions of RAW 264.7 lysates. (B) The effects of BS on a NF-κBreporter assay in HEK 293 cells that were activated by the addition of LPS. The luciferase activity was measured as relative light intensity, using a plate reader in the luminescence mode. BS, Fermented barley and soybean, LPS, Lipopolysaccharide. Figure S4. Immunohistochemicalstaining of IL-6 in sections from BS treated mouse colon. BS, Fermented barley and soybean. (PDF 563 kb) [file 12906_2016_1479_MOESM2_ESM.pdf]

Supplementary Figure S1

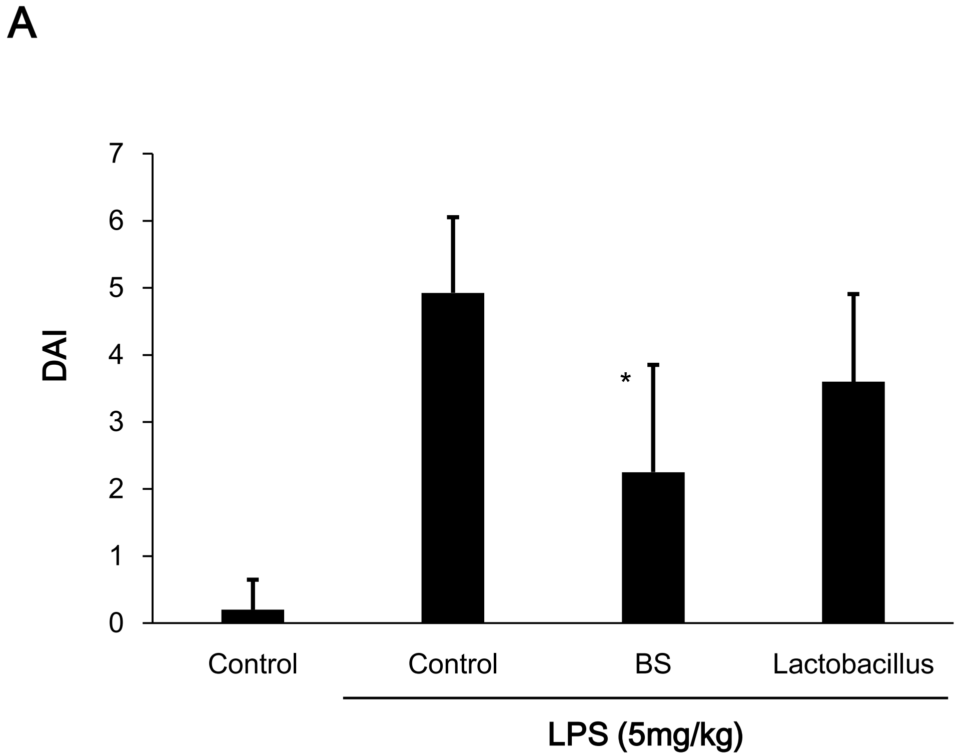

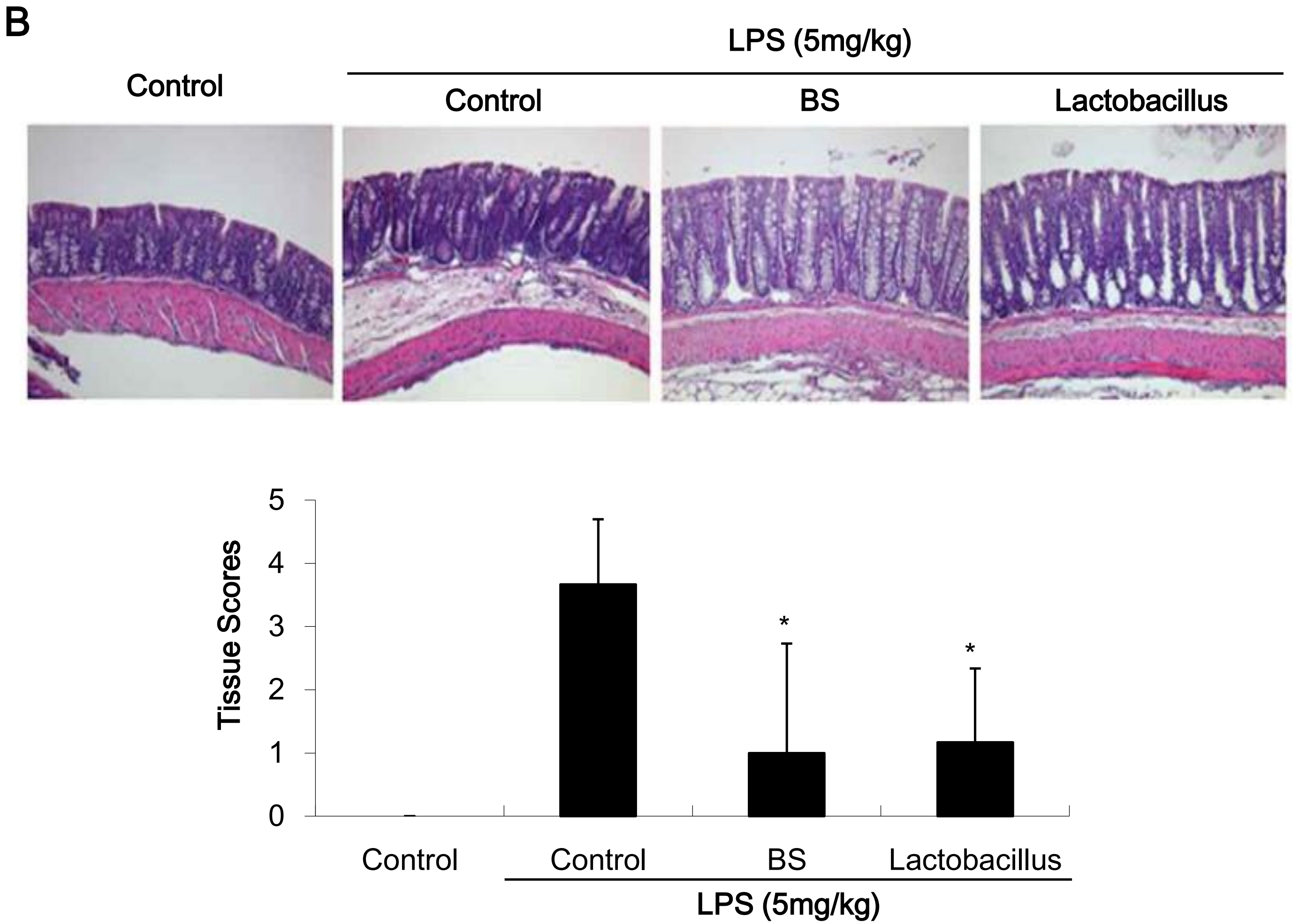

Figure S1. BS promotes recovery from LPS-induced colitis and inflammation in mice. (A) DAI of LPS (LPS, 5mg/kg of mouse by Intra-peritoneal injection) treated mice were scored. (B) Representative H&E stained histology from Control and BS-treated groups and statistical results. Mean value was significantly different from that of the LPS treated control group (* $P < 0.05$). BS, Fermented barley and soybean, DAI, disease activity index. LPS, Lipopolysaccharide

Supplementary Figure S2

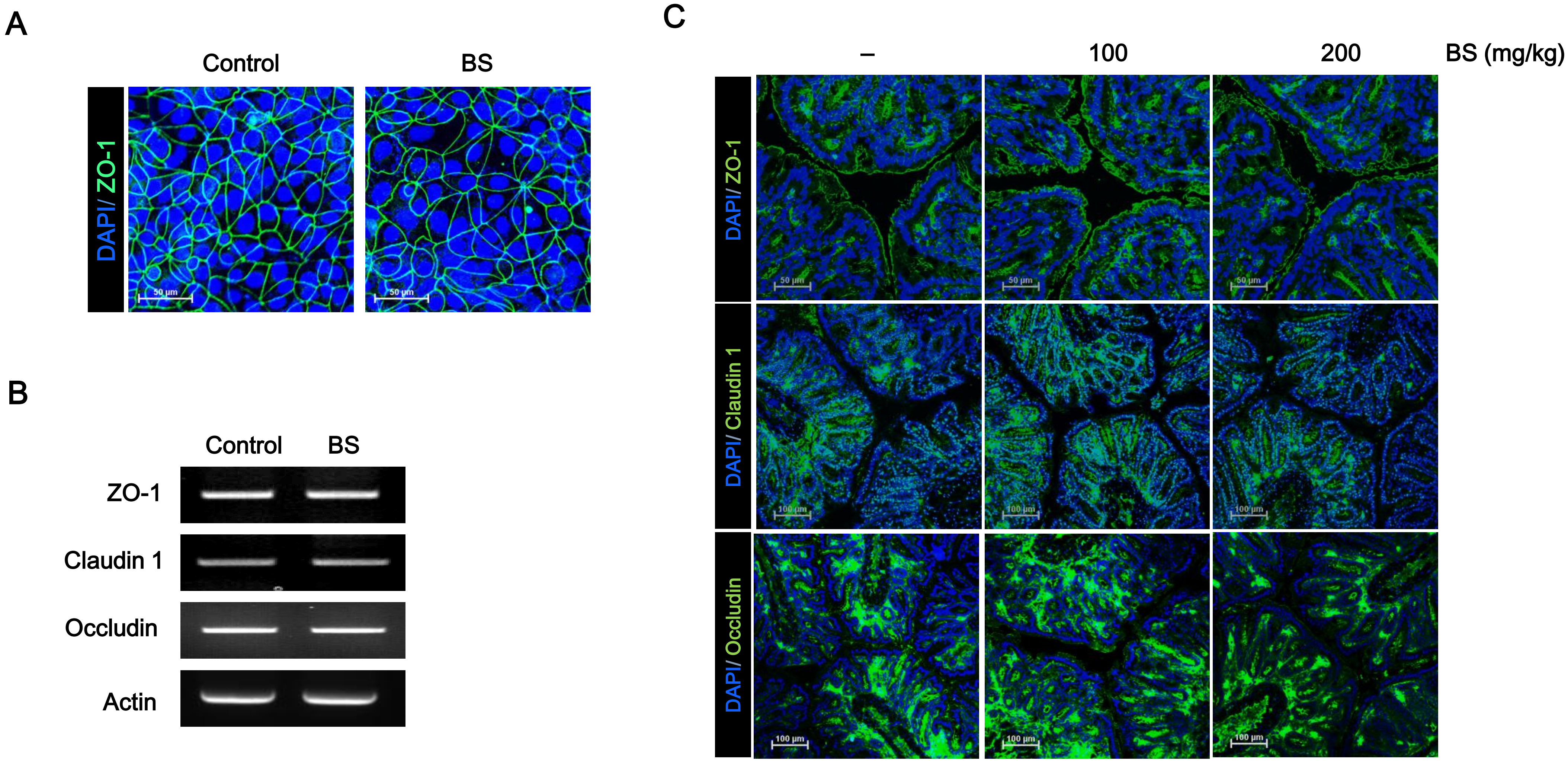

Figure S2. Basal intestinal barrier function in BS treatment. (A) Immunofluorescence of ZO-1 in Caco-2 cell monolayers incubated with BS for 48 h and images were collected by confocal microscopy. (B) RNA levels of ZO-1, Claudin 1, and Occludin in BS treated Caco-2 cells measured by semi-quantitative RT-PCR. (C) BS treated mouse colon tissues were used to determine ZO-1, Claudin 1 and Occludin distribution by immune fluorescence staining and images were collected by confocal microscopy. BS, Fermented barley and soybean,

Supplementary Figure S3

A

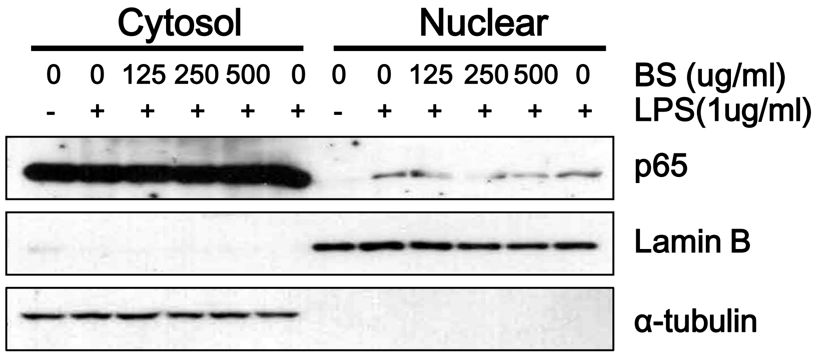

B

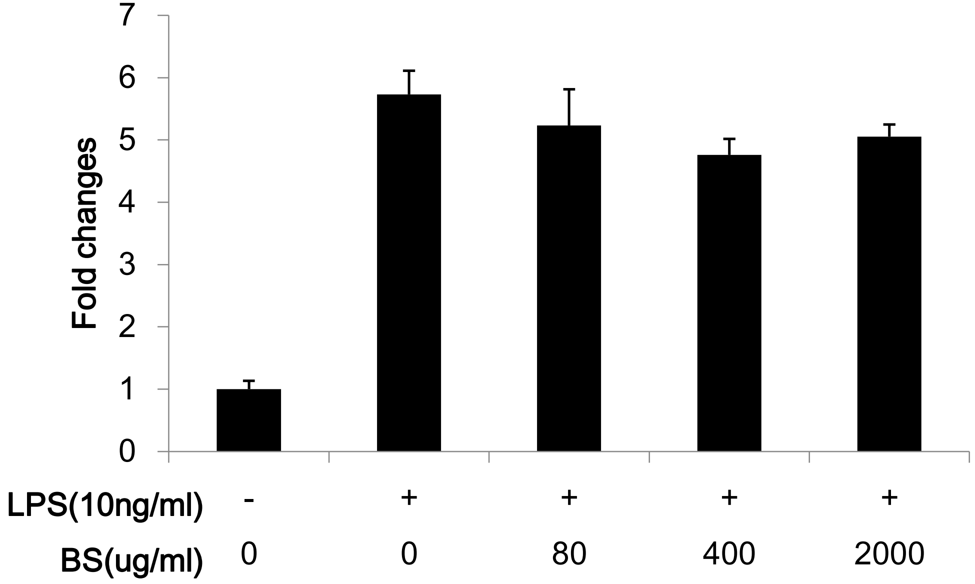

Figure S3. Effect of BS on the suppression of NF-κB activity. (A) Western blot analysis of p65 nuclear trans-localization levels in the protein fractions of RAW 264.7 lysates. (B) The effects of BS on a NF-κB reporter assay in HEK 293 cells that were activated by the addition of LPS. The luciferase activity was measured as relative light intensity, using a plate reader in the luminescence mode. BS, Fermented barley and soybean, LPS, Lipopolysaccharide

Supplementary Figure S4

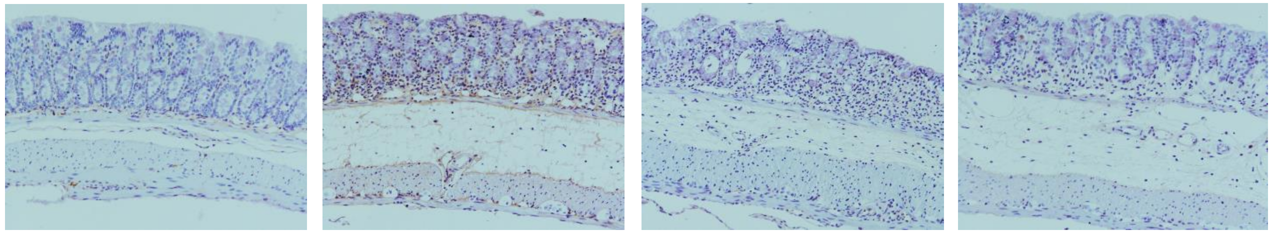

Figure S4. Immunohistochemical staining of IL-6 in sections from BS treated mouse colon. BS, Fermented barley and soybean
